# Complete cross strain protection against congenital cytomegalovirus infection requires a vaccine encoding key antibody (gB) and T-cell (immediate early 1 protein) viral antigens

**DOI:** 10.3389/fimmu.2025.1649656

**Published:** 2025-10-22

**Authors:** K. Yeon Choi, Yushu Qin, Nadia El-Hamdi, Alistair McGregor

**Affiliations:** Department of Microbial Pathogenesis & Immunology, Texas A&M University, Health Science Center, College of Medicine, Bryan, TX, United States

**Keywords:** cytomegalovirus, CMV, congenital CMV, gB, IE1, CMV vaccine, placenta, guinea pig

## Abstract

**Background:**

Cytomegalovirus is a leading cause of congenital disease, and multiple strains enable congenital CMV (cCMV) from both primary and non-primary infections. Therefore, a cross-strain protective cCMV vaccine is a high priority. Guinea pigs are the only small animal model for cCMV and guinea pig cytomegalovirus (GPCMV) encodes functional homolog proteins, including cell entry gB glycoprotein and non-structural immediate early 1 protein (IE1), which are essential for lytic infection. A gB vaccine antibody response fails to provide horizontal protection against highly cell-associated clinical GPCMV strain TAMYC compared to prototype strain 22122. Previously, a recombinant defective adenovirus (Ad) vaccine encoding IE1, a T cell antigen, provided high-level cCMV protection. In this study, we hypothesized that a combined Ad-based strategy encoding trimeric gB complex and IE1 (AdgB + AdIE1) could improve cross-strain protection against cCMV compared to a gB vaccine (AdgB).

**Methods:**

A preconception vaccine study was conducted to evaluate the immune response and ability of vaccines to provide cross-strain protection against cCMV. Seronegative female animals were assigned to three vaccine groups: Group 1 (AdgB), Group 2 (AdgB + AdIE1), and Group 3 (no vaccine). Animals were vaccinated following a previously defined protocol, and antibody ELISAs were used to evaluate the gB immune response (AD1, prefusion gB, and wild-type gB). Additionally, an IFNγ-ELISPOT assay was used to evaluate the IE1 T-cell response. During the second trimester, dams were challenged with GPCMV (22122 and TAMYC co-infection), and pregnancy proceeded to term. Viral loads in pup target organs (liver, lung, spleen, brain), blood, and placenta were evaluated.

**Results:**

Vaccinated dams elicited a higher gB neutralizing antibody response than that of experimentally infected convalescent animals. Antibodies recognized homolog AD1 gB domain as well as prefusion gB with a response surpassing that in GPCMV-infected convalescent animals. Group 2 dams also elicited a T cell response to IE1. Evaluation of viral load in pups demonstrated that the AdgB + AdIE1 vaccine reduced GPCMV transmission to below detectable limits compared to 91.7% in the unvaccinated group. In contrast, AdgB reduced cCMV transmission to 12% in the pups.

**Conclusion:**

Complete cross-strain cCMV protection is a significant milestone in this model and is achieved by the inclusion of an antibody response to trimeric gB and T-cell response to IE1. Importantly, gB and IE1 responses can synergize and increase protection against cCMV, unlike prior approaches with gB and pp65 tegument proteins.

## 1 Introduction

Human cytomegalovirus (HCMV) is a ubiquitous pathogen that establishes lifelong infections in immunosuppressed populations, including patients with transplants or AIDS. Additionally, HCMV can cross the placenta and cause congenital cytomegalovirus (cCMV) infection with symptomatic disease, causing vision and cognitive impairment as well as sensorineural hearing loss (SNHL) in newborns, and is associated with autism (1, 2). In Europe and the US, cCMV occurs in approximately 0.5%–1.2% of newborns, with up to 30% of hearing loss in children attributed to cCMV (3–5). Globally, over a million babies are born each year with cCMV (1), and a vaccine is considered a high priority (6). The greatest risk of cCMV is in mothers who acquire a primary infection during pregnancy (7). In the US, 50% of women of childbearing age are CMV seropositive (8). However, the development of a vaccine against cCMV is complicated by the existence of multiple strains of HCMV in the population, and convalescent natural immunity in seropositive individuals is insufficient to prevent reinfection (7). Therefore, cCMV can occur as a result of non-primary infection in women convalescent from the virus due to infection by a new strain and/or impaired immunity against the virus (1). Importantly, the level of cCMV related to non-primary infection is an underappreciated burden, especially on a global scale or in countries or regions with high endemic CMV levels, where cCMV levels can be as high as 5% (7, 8). Hence, the challenge of attaining a successful licensed vaccine against cCMV cannot be understated, with a requirement to greatly exceed the protective levels of natural convalescent immunity. Despite 50 years of vaccine research, a licensed cCMV vaccine remains an elusive goal, and antibody and T-cell responses to key target antigens are likely required.

HCMV species specificity complicates studies in preclinical animal models, which require the use of species-specific animal CMV. Additionally, there are only two animal models for cCMV (guinea pigs and rhesus macaques). Currently, no vaccine strategy has been evaluated against cCMV in a rhesus macaque non-human primate (NHP) model. Guinea pigs are the only small animal model for cCMV, with guinea pig cytomegalovirus (GPCMV) causing disease in newborn pups, similar to humans, including SNHL (9–12). The guinea pig has a hemomonochorial placenta structure similar to that of humans and a gestation period of approximately 70 days, which allows pregnancy studies to be evaluated in trimesters, with prenatal pup neuroanatomical development occurring almost completely *in utero* (13–15). The similarity of GPCMV and HCMV cell tropism, cell entry pathways, and functional homolog proteins as vaccine target antigens further emphasizes the importance of this model for congenital and systemic disease studies (12, 16–22). The majority of GPCMV research to date has focused on the prototype GPCMV 22122 strain. Despite the ability of the 22122 virus to cause cCMV, focusing on this strain for vaccine protection studies does not accomplish an important objective for a translational CMV vaccine model, i.e., cross-strain virus protection. A novel clinical strain of GPCMV (designated TAMYC) isolated from the salivary glands of a commercial animal was used by our laboratory to evaluate cross-strain vaccine efficacy, resulting in lower levels of protection in a non-pregnant animal model compared to studies with the 22122 strain (12, 19–21, 23–25).

A significant focus of CMV vaccine studies is directed towards the antibody response against viral glycoprotein complexes for cell entry (26–32). GPCMV has glycoprotein complexes similar to those of HCMV (gB, gH/gL/gO, and gM/gN), which are essential for the direct pathway of GPCMV infection of fibroblast cells and neutralization of target antigens (17, 20, 33–36). Additionally, both HCMV and GPCMV encode a glycoprotein pentamer complex (PC) that is necessary for the infection of non-fibroblast cells via an endocytic entry pathway (17, 22, 37, 38). In both HCMV and GPCMV, the gB glycoprotein is an immunodominant antibody target and is essential for the infection of all cells (17, 33, 39–41). A HCMV gB subunit vaccine, despite evoking high antibody titers, only provided approximately 50% efficacy in phase II clinical trials (42, 43). GPCMV gB subunit vaccine strategies in the guinea pig model fail to fully protect against cCMV and provide efficacy similar to that of human gB vaccine trials (44). However, the majority of GPCMV gB vaccine studies have only evaluated monomeric gB, and a homotrimeric gB vaccine strategy has the capacity to improve neutralizing antibody responses (20). Additionally, the inclusion of PC in a GPCMV vaccine improves virus neutralization in non-fibroblast cell lines and can enhance protection against cCMV (19, 34). However, this approach is less effective against highly cell-associated GPCMV (TAMYC) clinical strain (24).

In HCMV, convalescent patients produce T cell responses against two important viral proteins: viral tegument protein pp65 and non-structural IE1 protein (45). GPCMV encodes functional homologs of IE1 and pp65 (GP83) that are involved in innate immune evasion (21, 46). Both antigens induce cell-mediated immune responses in guinea pigs (34, 47, 48). Various pp65 (GP83) vaccine strategies against cCMV in the guinea pig model have had moderate levels of success, and the inclusion of both gB and pp65 in a vaccine approach did not improve cCMV vaccine efficacy against the 22122 strain (47, 49–51). Additionally, the pp65 antigen provided poor cross-strain protection against the clinical strain GPCMV (TAMYC) despite 100% identity in the pp65 amino acid sequence between strains (21). Recently, we demonstrated that GPCMV IE1 used in a recombinant Ad vaccine platform (AdIE1) provided high-level cross-strain protection against cCMV (48). In this report, we investigated the hypothesis that GPCMV cross-strain vaccine efficacy against cCMV could be improved compared to the prior AdIE1 vaccine by combining an immunodominant antibody target (trimeric capable GPCMV gB glycoprotein) with IE1 T cell antigen in a recombinant Ad vaccine strategy (AdgB + AdIE1) in comparison to a gB (AdgB) only vaccine or no vaccine control study group. Novel GPCMV gB antibody ELISAs were developed for prefusion gB and a homologous AD1 gB domain that is immunodominant for gB in HCMV. Vaccine strategies induced a higher antibody response to gB than experimental GPCMV (22122) inoculation/convalescent immunity with neutralizing antibodies effective against both prototype (22122) and clinical (TAMYC) strains of GPCMV. Additionally, the AdgB + AdIE1 vaccine induced a cell-mediated response to the IE1 unique protein sequence (GP123) in an IFNγ ELISPOT assay. In a preconception vaccine strategy, the AdgB + AdIE1 combined vaccine provided the first successful approach for complete cross-strain protection against cCMV in this preclinical animal model. The AdgB + AdIE1 vaccine group had no detectable virus in pup litters compared to the detectable virus in both the no-vaccine control group and standalone AdgB vaccine group. The results demonstrate the combined importance of IE1 and gB as key antigens for a successful protective vaccine strategy against cCMV, evoking both humoral (gB) and cell-mediated (IE1) immune responses against CMV.

## 2 Materials and methods

### 2.1 Cells, viruses, oligonucleotides, and genes

GPCMV (strain 22122, ATCC VR682) was propagated in various cell lines. Guinea pig fibroblast lung cells (GPL; ATCC CCL 158) and renal epithelial cells (REPI) were used for specific tropism studies, as previously described (17, 18). Both clinical strain GPCMV TAMYC (25) and 22122 strain were expanded from separate guinea pig salivary gland stocks and exclusively propagated on REPI cells for the maintenance of fully tropic virus stocks and animal challenge studies (17). Recombinant defective adenovirus (Ad5) vectors (E1 and E3 deleted) were previously described (20, 48) encoding full-length gB (AdgB) or IE1 cDNA (AdIE1) with ORFs under HCMV IE enhancer control with a 3’ SV40 polyA sequence. High-titer CsCl gradient-purified recombinant defective adenovirus stocks (10^12^ TDU/ml) were generated by Welgen Inc. (MA). All oligonucleotides were synthesized by Sigma Genosys (The Woodlands, TX, USA). Synthetic codon-optimized genes were developed for (1) prefusion gB (prefgB) and (2) gB AD1 domain as a fusion protein with gB(AD1) expressed at the C-terminus of GST, designated GST-gB(AD1). Based on the alignment of GPCMV gB with HCMV gB protein amino acid sequences, a GPCMV prefgB was generated by gene synthesis (Genscript) incorporating the removal of the furin site and additional specific amino acid substitutions equivalent to those made in the HCMV gB ectodomain to generate a locked version of GPCMV prefgB (52). The ORF also incorporated a C-terminal FLAG epitope tag. The GPCMV prefgB was cloned into the pcDNA3.1(+) vector (Invitrogen) under HCMV IE enhancer promoter control to enable transient expression in plasmid-transfected cells (see *ELISA* section).

### 2.2 Ethics

Guinea pig (Hartley) animal studies were performed under the IACUC (Texas A&M University). All study procedures were carried out in strict accordance with the recommendations in the “Guide for the Care and Use of Laboratory Animals of the National Institutes of Health.” Animals were observed daily by trained animal care staff, and those that required care were referred to the attending veterinarian for immediate care or euthanasia. Terminal euthanasia was performed by lethal CO_2_ overdose followed by cervical dislocation in accordance with the IACUC protocol (Texas A&M University) and NIH guidelines. The animal chamber was filled with 100% CO_2_ at a rate of 30%–70% of the chamber volume per minute with CO_2_, added to the existing air in the chamber. This is appropriate for achieving a balanced gas mixture to fulfill the objective of rapid animal unconsciousness with minimal distress. Animals purchased from Charles River Laboratories were verified as seronegative for GPCMV by toenail clip bleeding and anti-GPCMV ELISA of sera, as previously described (33). Animal studies were conducted to determine (1) GPCMV (TAMYC strain) pathogenicity in seronegative or seropositive (22122 strain) animals; (2) immune response to natural convalescent immunity (GPCMV 22122 strain); (3) immune response of candidate vaccines (AdgB or AdgB + AdIE1); (4) vaccine efficacy against congenital GPCMV challenge (22122 and TAMYC strains) and protection of pups in the vaccine group compared to the no-vaccine control group.

### 2.3 Animal studies

(A) Experimental single inoculation of GPCMV to establish immunity equivalent to natural convalescent immunity and protection from GPCMV infection. GPCMV (22122) convalescent immunity in animals and cross-strain protection against TAMYC strain challenge subcutaneous inoculation (SQ) route. Animal studies were performed to evaluate protection against the TAMYC strain GPCMV challenge in animals convalescent from the 22122 strain. A seropositive animal group was created by subcutaneous GPCMV inoculation of seronegative female guinea pigs (n = 12). They received the GPCMV strain 22122 at 10^5^ pfu (SQ), and 3 months later, seroconversion was evaluated using an anti-GPCMV antibody ELISA screen and designated as seropositive convalescent animal group. Pooled sera from seropositive (22122) convalescent animals were used for additional evaluation of immune responses to specific viral glycoprotein complexes (**Supplementary Figure S1**) and virus neutralization studies. A second group of animals (n = 12) was verified as negative for GPCMV by anti-GPCMV ELISA and used as the seronegative animal group. Animals in both groups were challenged with the TAMYC strain GPCMV (10^5^ pfu, SQ) on day 0. At 4, 8, 12, and 27 days post-infection (DPI), three animals per group were euthanized to evaluate the viral load in target organs and blood via real-time PCR assay, as previously described (23). (B) AdgB or AdgB + AdIE1 preconception vaccine protection against congenital GPCMV (TAMYC and 22122 strains). Seronegative female guinea pigs were randomly assigned to two groups. Group 1 (AdgB vaccine group; n = 17) or Group 2 (AdgB + AdIE1 vaccine group; n = 13) were vaccinated with SQ with the corresponding vaccine (10^8^ TDU) and boosted 4 and 8 weeks post original vaccine with equivalent dosage. Group 3 animals (n = 10) were assigned to the no-vaccine control group. At 4 weeks after the last vaccination, dams were paired with seronegative males for mating. The control group was similarly paired for mating purposes. Dams were confirmed to be pregnant by palpation at approximately 20–25 days of gestation. At the late second trimester/early third trimester, pregnant animals in all groups were challenged with both strains of wild-type GPCMV (22122 and TAMYC strains) in separate SQ injections (10^5^ pfu GPCMV/injection) into opposite flanks, and the animals were allowed to go to term. Subsequently, the viral load in the target organs (liver, lung, spleen, and brain) and blood of live-born or stillborn pups was evaluated using real-time PCR. Placental tissue, when available, was also evaluated for viral load (approximately 75% recovery of placenta from all groups).

### 2.4 Real time PCR assay

Tissues were collected from the euthanized guinea pigs to determine the viral load. For tissue DNA extraction, FastPrep 24 (MP Biomedicals) was used to homogenize the tissues as a 10% weight/volume homogenate in Lysing Matrix D (MP Biomedicals). DNA was extracted using the QIAcube HT (Qiagen) according to the manufacturer’s tissue protocol instructions. Viral load was determined by real-time PCR on a LightCycler 480 (Roche Applied Science), as previously described (17, 34). Primers and hydrolysis probes were designed using the LightCycler Probe Design2 program to amplify a product from the GPCMV viral polymerase subunit *GP44* gene: forward primer 5’TCTCCACGGTGAAAGAGTTGT; Reverse primer 5’GTGCTGTCGGACCACGATA; hydrolysis probe 5’FAM-TCTTGCTCTGCAGGTGGACGA-BHQ1. Data were analyzed using the LightCycler Data Analysis Software (version 1.5.1; Roche). A standard curve was generated using serial dilutions of the GPCMV 22122 *GP44* plasmid at known concentrations for quantification and assay sensitivity. The same standard curve was generated using TAMYC *GP44* plasmids to demonstrate that the Universal GP44 primer-probe set could detect both strains. The sensitivity of the assay was determined to be two copies per reaction. Viral load was expressed as copy number/mg tissue or copies/ml of blood. The results were calculated as the mean value of all positive results. Positive triplicates were considered as positive. Assays were repeated for those with any wells below the level of detection. Results were considered positive if at least 2/3 wells were positive on repeated runs. Wells with extrapolated data below the level of detection were not included in the mean calculation as positive results.

### 2.5 ELISA and Western blot assays

#### 2.5.1 ELISA

Anti-GPCMV ELISA was carried out as previously described (33) to determine GPCMV sero-status of commercially obtained guinea pigs (Charles River) as commercial animal colonies are not negative for GPCMV. Specific glycoprotein complex ELISAs (gB, gM/gN, gH/gL, and PC) were performed as previously described (19) using positive coating antigens derived from renal epithelial cell monolayers transduced with recombinant replication-defective adenovirus (Ad) vectors expressing specific viral glycoprotein complexes or recombinant Ad vectors expressing GFP as a negative coating antigen (17, 33, 34). In the case of gM/gN, codon-optimized synthetic genes in mammalian plasmid expression vectors were used instead (19, 33). Novel gB glycoprotein assays were developed for prefgB and gB(AD1) ELISAs. For prefgB, a synthetic prefusion gB mammalian expression plasmid (pcDNA3.1(+), Invitrogen) was used to express prefgB in REPI cells to generate a coating antigen, following a similar protocol for gM/gN ELISA (19, 33). The expression of prefgB was verified using Western blotting (**Supplementary Figure S2**). A homologous AD1 domain was previously identified between HCMV gB and various animal CMV gB proteins based on BLAST alignment (53). Alignment of the GPCMV gB and HCMV gB ORFs encoding the AD1 region is shown in **Supplementary Figure S3A**. The minimal AD1 region was extended for GPCMV gB with additional flanking N- and C-terminal amino acids to match the extended AD1 sequence used to express a commercial HCMV gB(AD1) protein, as a GST-gB(AD1) fusion protein (Sigma-Aldrich). A synthetic GST protein ORF encoding the C-terminal fused GPCMV gB(AD1) region (gB amino acid codons 524-646) was cloned into a bacterial expression vector and expressed as a recombinant protein (Genscript). The recombinant protein was purified using GST affinity chromatography for use in gB(AD1) ELISA studies. For gB(AD1) ELISA, the purity of the recombinant gB(AD1) protein was verified using SDS-PAGE, Coomassie gel staining, and Western blotting (**Supplementary Figure S3**). Both antigens were determined for optimal plate coating concentrations: A) 2 ug/ml prefgB; B) 3 ug/ml gB(AD1). The net OD (absorbance at 450 nm) was obtained by subtracting the OD of Ag− from that of Ag+. ELISA reactivity was considered positive if the net OD was ≥0.2, as determined using GPCMV-negative serum. Assays on pooled sera were conducted a minimum of three times, and the mean titer was determined. In addition, varying coating concentrations of the control carrier recombinant GST protein (Genscript) were tested for background using both GPCMV seropositive and seronegative control sera (**Supplementary Figure S4**).

#### 2.5.2 Western blots

Western blotting was performed on cell lysates separated by 10% SDS-PAGE under denaturing conditions as previously described (17, 33). For western blots, anti-epitope tag primary antibodies were used at 1/1,000: mouse anti-FLAG M2 (Sigma) (17, 19) and mouse anti-GST (Genscript). Secondary antibodies: Anti-mouse HRP-linked secondary antibodies for western blot (Cell Signaling) were used at ½,000 (19). The FLAG epitope was used to detect prefgB protein expression in transduced GPL cell monolayers. GST was used to detect the gB(AD1)-GST-tagged fusion protein. PrefgB was evaluated under denaturing and non-denaturing conditions. For evaluation under non-denaturing conditions, prefgB was analyzed as previously described (20), with gB protein expression (monomeric) and multimerized protein both detected (**Supplementary Figure S2**).

### 2.6 IE1 guinea pig interferon gamma enzyme-linked immunospot assay

Guinea pig IE1 IFNγ ELISPOT assays were performed following a previously described protocol using freshly isolated splenocytes and GPCMV GP123 peptide pools (19). Anti-guinea pig IFNγ monoclonal antibodies (V-E4 and N-G3) were previously characterized (54). Briefly, PVDF membrane 96 well plates were coated with guinea pig IFNγ capture antibody, V-E4, and incubated overnight at 4 °C, blocked, and freshly isolated splenocytes were added before being exposed to GPCMV GP123 15mer peptide pools and incubated for 18 h. Biotinylated detection antibody, N-G3 was added before streptavidin-AP conjugate (R&D Systems) and then detected with BCIP/NBT (Life Technologies). Membranes were dried before spots were counted on ImmunoSpot S6 (CTL). Final counts were calculated based on spot-forming cells (SFC) per 10^6^ cells after background spots (cells only without any stimulation) were subtracted. Assay controls included Con A (10 µg/ml) as positive stimulation control, cells only control, DMSO control (peptide background), nonspecific peptide control, and media only control. Control animals included GPCMV seropositive (n = 3) and uninfected GPCMV seronegative animals (n = 2) prior to peptide stimulation.

GP123 peptide pools. Eighty-seven 15mer peptides (overlapping by 11 amino acids) covering the unique IE1 protein-coding sequence GP123 were generated by GenScript. The peptide pools included 8–10 peptides, generating a total of 19 pools (**Supplementary Tables 1, 2**). Each pool was tested as described above (GP IFN-γ ELISPOT assays) to determine the most reactive pools.

### 2.7 Statistical analysis

All statistical analyses were conducted using GraphPad Prism (version 7) software. Fisher’s exact test and unpaired Student’s t test were used, with significance set at p  <0.05 or as specified in the figure legends.

## 3 Results

### 3.1 GPCMV convalescent immunity (22122) does not prevent reinfection (TAMYC strain)

**Figure 1:**
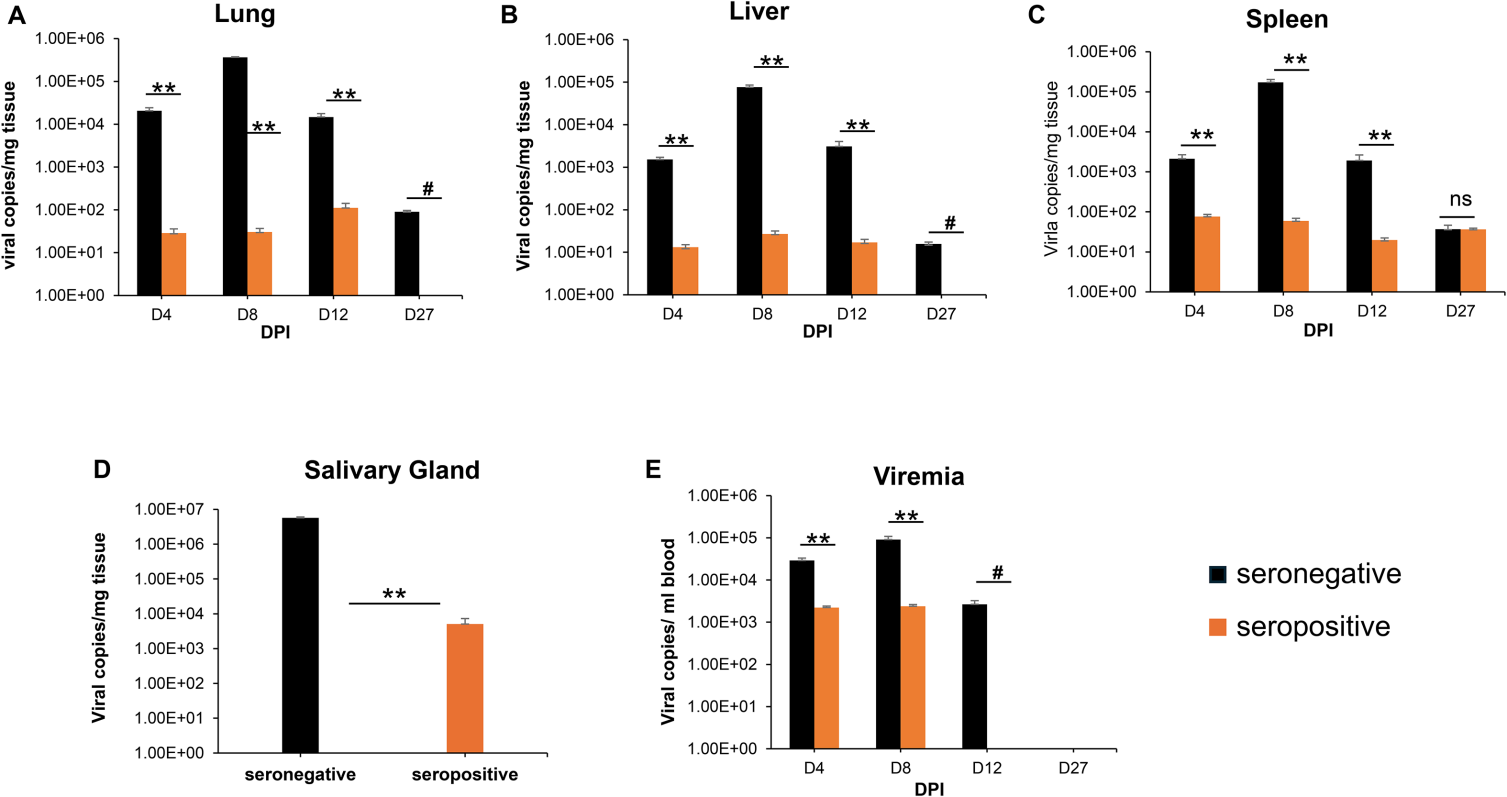
GPCMV (22122 strain) convalescent immunity does not protect against heterologous GPCMV (TAMYC strain) challenge. Seronegative animals (n = 12) were inoculated once with the 22122 strain GPCMV (10^5^ pfu, SQ). After seroconversion was verified, the animals were designated as seropositive (orange). At 3 months post original infection, animals were challenged with the TAMYC strain of GPCMV (10^5^ pfu, SQ). A control group (n = 12) of seronegative animals (black) was similarly challenged with the TAMYC strain GPCMV. At 4, 8, 12, and 27 days-post infection (DPI), three animals per group were evaluated for viral load in target organs using real-time PCR of tissue-extracted DNA. The viral load was plotted as the mean number of viral genome copies/mg of tissue. Salivary gland tissues were evaluated only on day 27. **(A)** Lung, **(B)** liver, **(C)** spleen, and **(D)** salivary gland. Viremia **(E)** at 4, 8, 12, and 27 dpi is plotted as the mean number of genomic copies/ml blood. Statistical analysis was performed using an unpaired Student’s t-test; **p < 0.001; ns, not significant; ^#^viral load in the CMV^+^ group below the level of detection.

To demonstrate the limited ability of GPCMV-infected (22122 strain) convalescent animals to provide cross-strain protection against horizontal (SQ) challenge (TAMYC strain), animals were initially infected with the 22122 strain of GPCMV to establish convalescent immunity. Seronegative animals (n = 12) were inoculated with a single injection (10^5^ pfu, SQ) of GPCMV (22122 strain) to mimic a natural infection. Animals were evaluated for anti-GPCMV seropositive status as well as evaluated for immune response to specific viral glycoprotein complexes (gB, gM/gN, gH/gL, and PC) at 3 months post-infection. All animals seroconverted to GPCMV seropositive status (mean anti-GPCMV titer 5120). The animals also had an immune response to all GPCMV viral glycoprotein complexes (**Supplementary Figure S1**). Next, the animals were challenged with 10^5^ pfu of GPCMV (TAMYC) by the SQ route. At 4, 8, 12, and 27 dpi, animals were randomly euthanized (n = 3), and the viral load (lung, liver, spleen, and blood) was evaluated. Additionally, salivary gland tissue was also evaluated for viral load at 27 dpi. Results (**Figure 1**) were compared to a previous historical study of the TAMYC strain virus challenge with the same virus stock in seronegative animals infected with an identical dose of virus (19) and demonstrated that the TAMYC strain (22122) seropositive status of animals. However, the viral load at all time points was significantly reduced compared to previous TAMYC virus dissemination in seronegative animals, except in the D27 spleen, which was not significant (**Figure 1**) (25). Overall, the results demonstrated the limited ability of 22122 strain convalescent immunity to prevent viremia and dissemination of the subsequent virus challenge by the heterologous TAMYC strain.

### 3.2 Preconception Ad vaccine (AdgB or AdgB + AdIE1) immune response in animals

**Figure 2:**
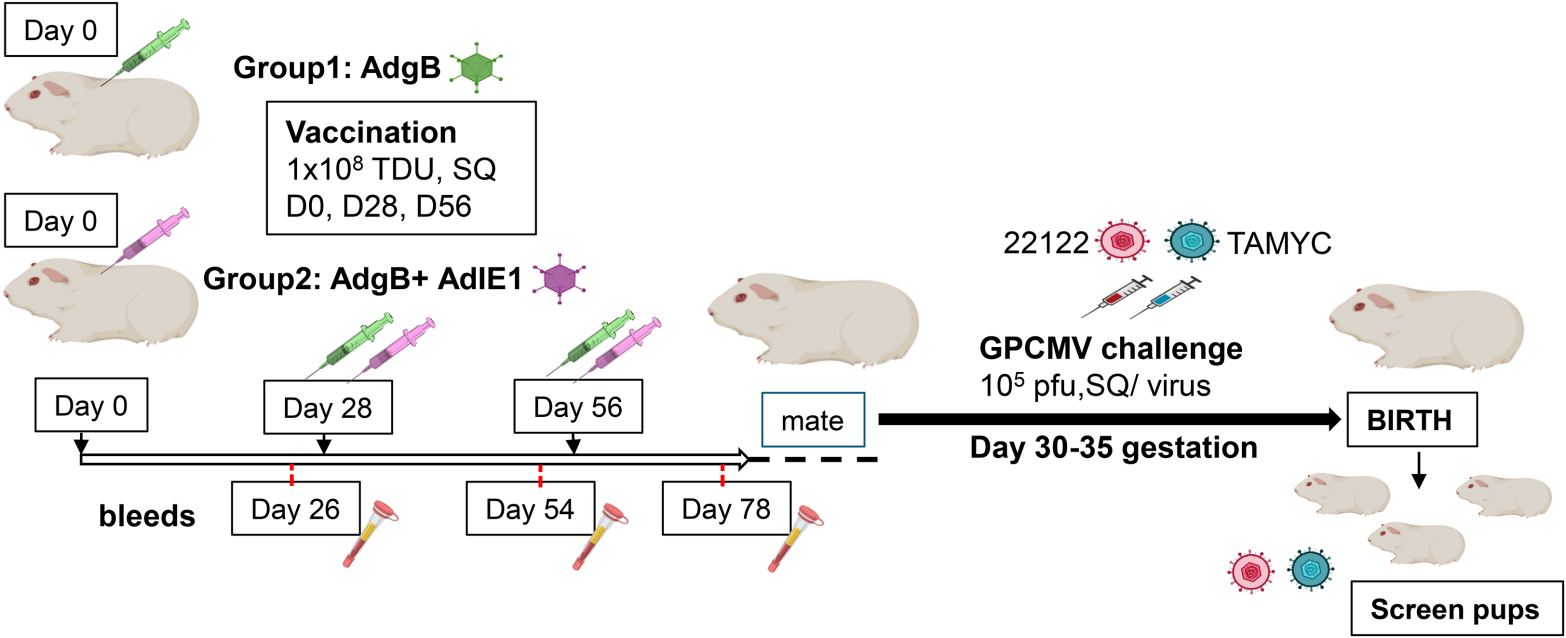
Schematic overview of the preconception vaccine study and GPCMV congenital virus challenge with dual viral strains (22122 and TAMYC). Seronegative dams: Group 1 (AdgB, n = 17) or Group 2 (AdgB + AdIE1, n = 13) were vaccinated three times (10^8^ TDU) via the SQ route on days 0, 28, and 56. Serum was collected 3 to 4 weeks after each vaccination (days 26, 54, and 78). The dams were mated, and during the late second trimester of pregnancy, the animals were challenged with both 22122 and TAMYC strains (10^5^ pfu/virus, SQ route) then followed to term. The viral load in the target organs (liver, lung, spleen, and brain) of live-born or still-born pups was evaluated using real-time PCR. Placental tissue, when available, was also evaluated for viral load. A control group of unvaccinated pregnant dams (n = 10) was similarly challenged with GPCMV, and pup tissues were evaluated for viral load.

**Figure 3:**
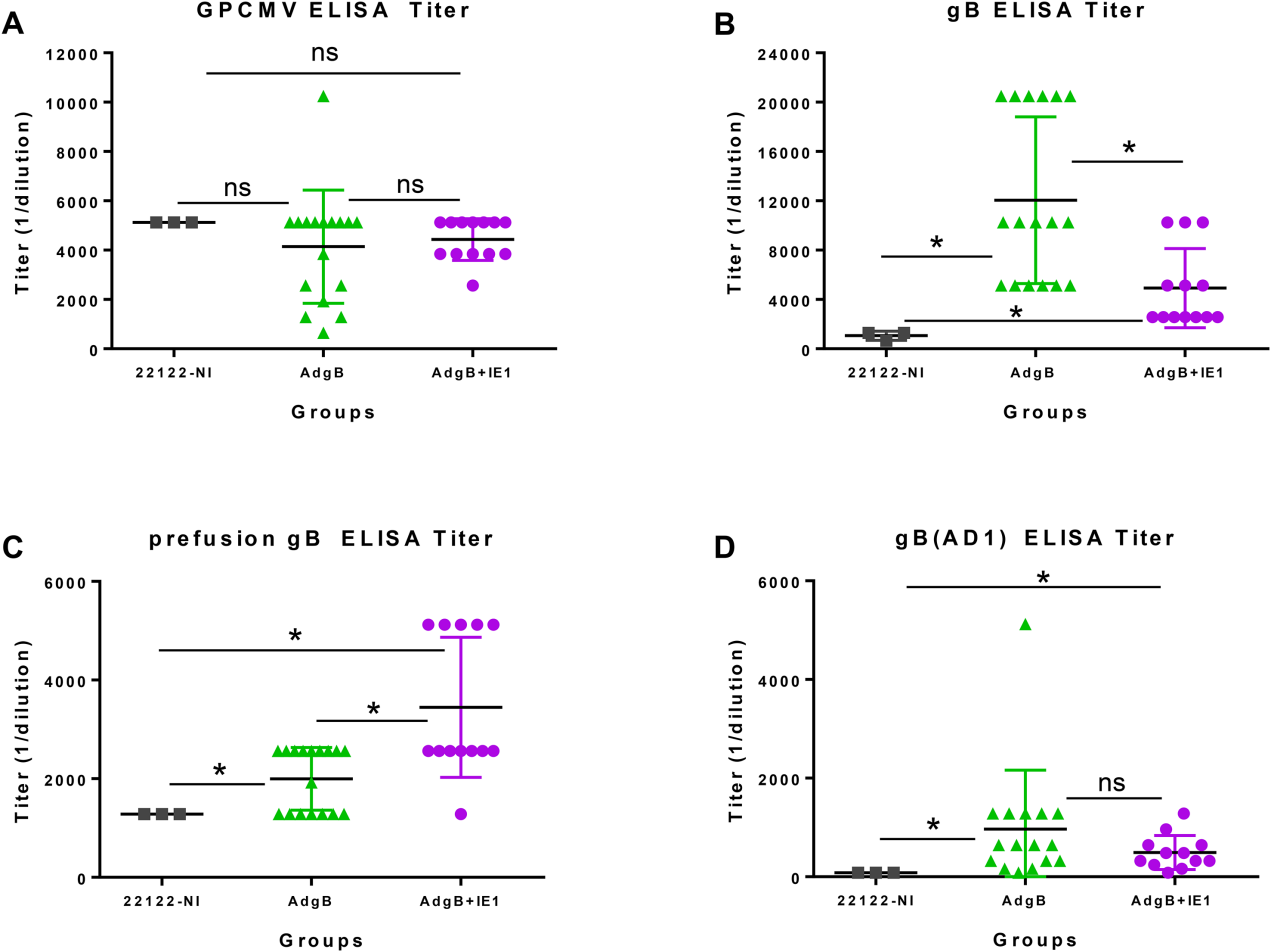
Antibody immune responses to AdgB or AdgB + AdIE1 vaccines compared to the convalescent immunity of wild-type GPCMV-naturally infected (22122-NI) animals. Individual animal sera from AdgB (green) or AdgB + AdIE1 (purple) vaccinated animals were compared to pooled convalescent sera of naturally infected GPCMV (black) animals for antibody titers to specific target ELISAs: **(A)** anti-GPCMV; **(B)** anti-gB; **(C)** anti-prefusion gB; or **(D)** anti-gB(AD1). Pooled 22122-NI serum assays were repeated three times. The mean value for each group is represented by a black horizontal line. Statistical analysis was performed using an unpaired Student’s t-test, *p <0.05; ns, not significant.

Subsequently, the ability of Ad-based vaccines to trimeric capable gB and IE1 antigens were evaluated for their ability to provide cross-strain protection against cCMV. An outline of this study is shown in **Figure 2**. Seronegative dams were randomly assigned to two vaccine groups: Group 1 (AdgB), n = 17; Group 2 (AdgB + AdIE1), n = 13. At days 0, 28, and 56, animals were vaccinated SQ with a specific CMV vaccine candidate according to the assigned group (vaccine dosage 10^8^ Transduction Units (TDU)/shot per Ad vector) with bleeds at days 26, 54, and 78 for evaluation of anti-GPCMV antibody response. At day 78 post-initial vaccination, the antibody immune response was characterized in depth using a series of ELISA assays for individual animals: anti-GPCMV; anti-gB(wt); prefusion gB; and gB (AD-1 domain). The results were compared between groups 1 and 2 and with pooled convalescent sera from single shot 22122 infected animals (**Figure 3**). The pooled sera from single-shot GPCMV-infected animals had similar anti-GPCMV mean titers to vaccine groups 1 and 2, but the anti-GPCMV titers for individual animals in the AdgB vaccine group were more varied than those in the AdgB + AdIE1 group, despite similar overall mean titers (**Figure 3A**). The anti-gB response (trimeric capable gB) was on average higher for group 1 animals (mean titer 12047) than for the AdgB + AdIE1 group (mean titer 4923), which was statistically significant (**Figure 3B**); however, these titers were not uniformly higher for all animals in group 1 than in group 2. Both groups had statistically higher anti-gB titers than pooled sera from single-shot 22122 strain-infected animals.

Prefusion gB is a newly defined antigen for HCMV (52) and has not been previously evaluated in GPCMV. Based on the BLAST alignment between the predicted amino acid sequences of HCMV gB and GPCMV gB, a locked version of GPCMV prefusion gB was generated by incorporating specific codon changes used for the generation of HCMV prefusion gB and removal of the furin cleavage site (52). A synthetic codon-optimized gene encoding prefusion gB (prefgB) was generated (Genscript) and cloned into a mammalian expression plasmid which was used to generate the ELISA coating prefgB antigen as described in the *Materials and methods* section. Prefusion gB expression in plasmid-transfected cells was verified by Western blot assay, which detected both monomeric and multimeric forms of prefgB (**Supplementary Figure S2**). Evaluation of the immune response to prefusion gB indicated that group 2 animals had a higher titer (mean titer 3,446) than group 1 animals (mean titer 1,995), and the difference was significant (**Figure 3C**). Both vaccine groups had significantly higher anti-prefusion gB titers than the pooled sera from 22122 infected animals (mean titer 1,280) (**Figure 3C**).

The gB AD1 region is considered the immunodominant domain in HCMV gB but has not been previously evaluated for GPCMV in prior GPCMV gB vaccine studies. A bacterial expression plasmid encoding the GST-gB(AD1) fusion protein was constructed based on the BLAST alignment of the HCMV gB AD1 domain with the GPCMV gB ORF (**Supplementary Figure S3A**) (53). A synthetic gene encoding an extended GPCMV gBAD1 fused to the C-terminus of the GST carrier protein was generated as described in the *Materials and methods* section. The GST-gB(AD1) protein was expressed in bacteria, and the recombinant protein was purified using GST affinity column chromatography and verified by Coomassie-stained SDS-PAGE (**Supplementary Figure S3B**) and Western blot analysis (**Supplementary Figure S3C**) prior to use as the gB(AD1) ELISA coating antigen. A control ELISA with recombinant GST protein (Genscript) lacked activity with GPCMV seropositive and seronegative sera, demonstrating a lack of preexisting antibodies to GST protein in animals (**Supplementary Figure S4**). Anti-gB(AD1) ELISA immune responses were more tightly clustered for individual animals within the vaccine groups, with only one animal titer relatively high in group 1 (5120) compared to the mean value of 965. However, the difference in mean titer between groups 1 and 2 animals was not significant (**Figure 3D**). Both vaccine groups produced a significantly higher anti-gB(AD-1) response than the 22122 strain convalescent sera (mean titer 80) (**Figure 3D**). Overall, based on ELISA studies, the biggest difference between the vaccine groups was in the immune response to prefusion gB, which was highest in group 2 animals. Additionally, the trimer gB ELISA titer was highest in group 1 animals by greater than 2-fold.

**Figure 4:**
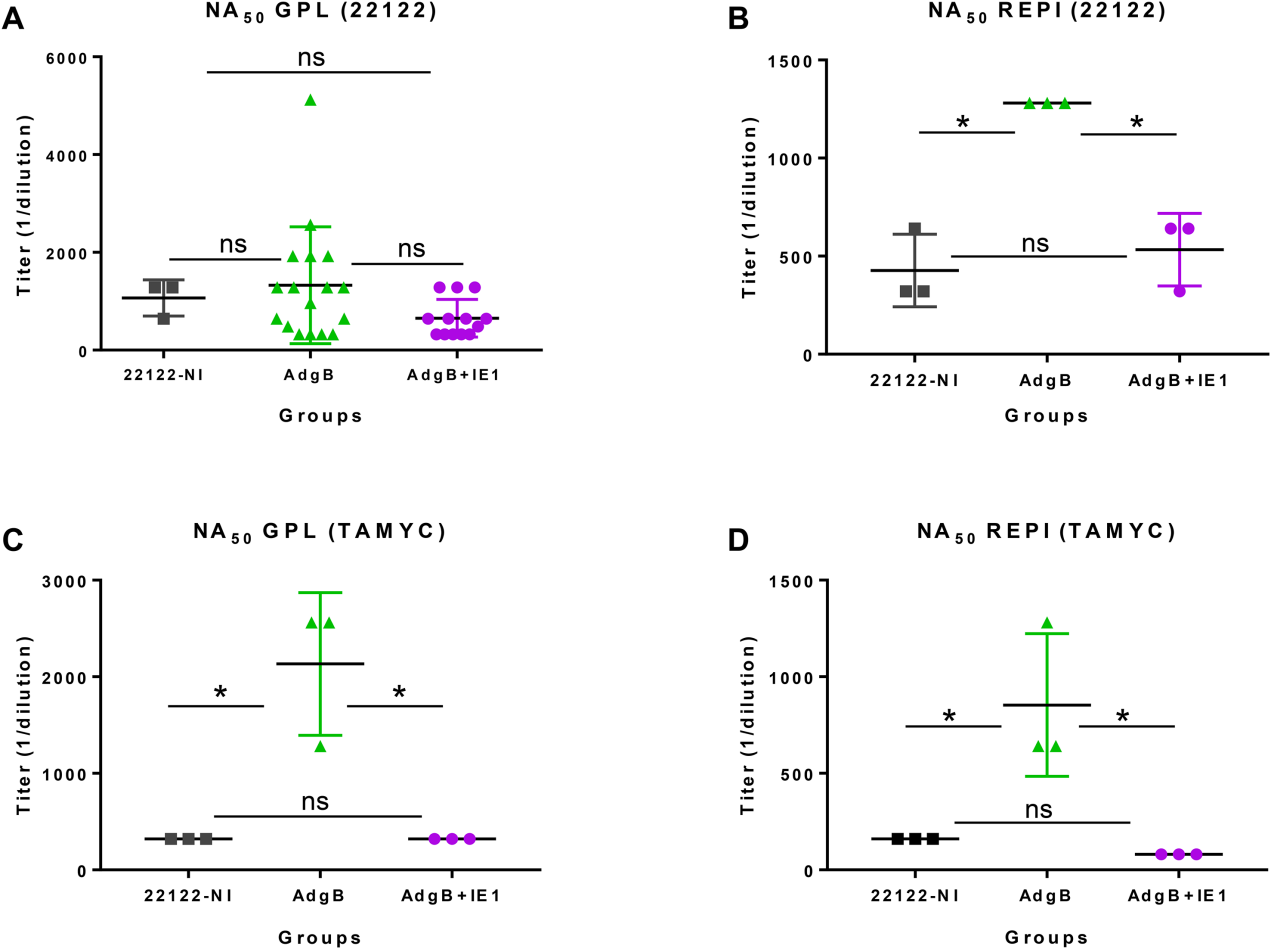
GPCMV neutralization by anti-AdgB or anti-AdgB + AdIE1 animal sera compared to GPCMV (22122) natural convalescent sera (22122-NI). Individual animal sera of AdgB (green) or AdgB + AdIE1 (purple) vaccinated animals was compared to pooled sera of GPCMV (22122) convalescent sera (black) animals for neutralization of 22122 or TAMYC strain GPCMV on different cell lines: **(A)** neutralization of 22122 on GPL fibroblasts; **(B)** neutralization of 22122 on epithelial cells (REPI); **(C)** neutralization of TAMYC virus on GPL cells; **(D)** neutralization of TAMYC virus on epithelial cells. Pooled 22122-NI sera assays were repeated three times. Mean value from each group represented by black horizontal line. Statistical analysis was performed using an unpaired Student’s t-test, *p <0.05; ns = not significant. Assays were carried out with pooled sera within different groups, except for **(A)**, where vaccine sera from individual animals from both vaccine groups were evaluated.

Next, sera from vaccinated animal groups or convalescent sera (22122 infected animals) were evaluated for neutralizing antibody titers in both fibroblast (GPL) and epithelial (REPI) cells for GPCMV strains 22122 and TAMYC (**Figure 4**). Neutralization assays were conducted using the same sera collected 78 days after the initial vaccination. Evaluation of GPL NA_50_ (22122 strain) for individual sera from each vaccine group compared to pooled convalescent sera did not demonstrate any significant difference between groups, but individual NA_50_ titers for group 1 animals were more widely spread than those for group 2 animals (**Figure 4A**). Pooled sera within groups were subsequently used for the evaluation of NA_50_ (22122) on epithelial cells, where the group 1 vaccine had a significantly higher NA_50_ titer than group 2 or convalescent 22122 infected animal sera (**Figure 4B**). There was no significant difference in NA_50_ titers between the group 2 animals and the 22122 convalescent sera. Evaluation of NA_50_ (TAMYC) resulted in a higher NA_50_ titer for the AdgB (group 1) vaccine group than for the other serum groups on both fibroblasts and epithelial cells (**Figures 4C, D**). Overall, the results suggest that group 1 (gB vaccine) sera had slightly better neutralizing capability against both strains of the virus.

**Figure 5:**
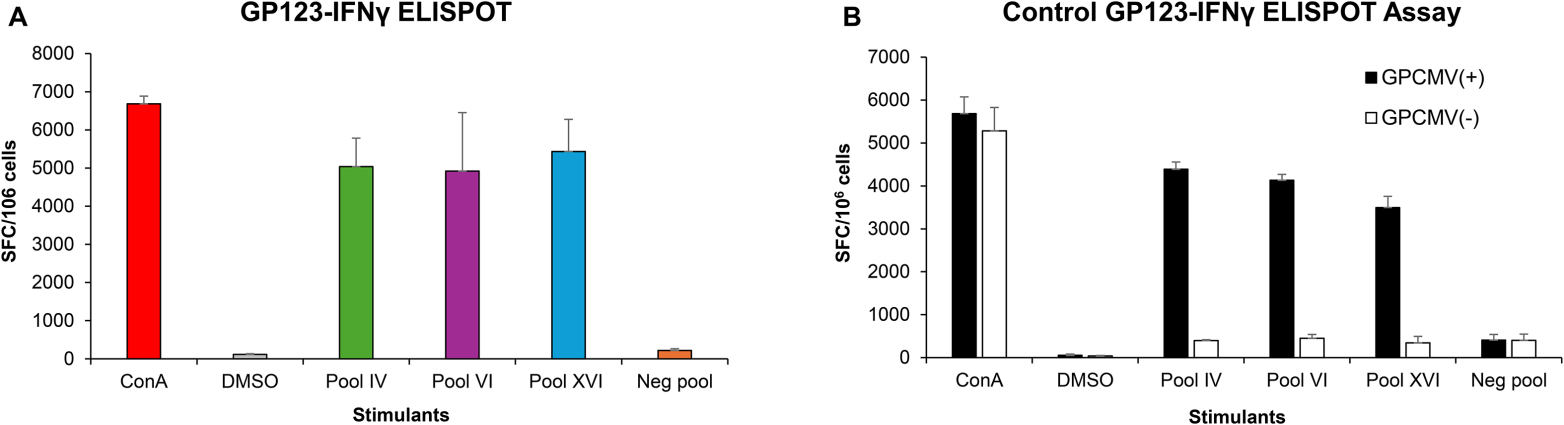
T cell response to GPCMV IE1. Guinea pig-specific IFNγ ELISPOT was performed using pools of overlapping peptides for GP123. **(A)** AdgB + AdIE1 vaccinated animals. Three GP123-reactive peptide pools, IV, VI, and XVI (green, purple, and blue bars, respectively) were identified to react with primed splenocytes from three vaccinated animals and are shown as mean values. ConA assay positive control (red), DMSO non-specific background control (gray), and non-reactive negative pool (orange) were also included in each assay plate, and mean values were represented. **(B)** GPCMV-seropositive (black) and GPCMV-seronegative (white) animals. Splenocytes from animals were assayed with the GP123 peptide pool as described for **(A)**, and the mean values are represented for the same reactive and non-reactive pools. ConA assay positive controls and DMSO non-specific background controls were included in each assay plate. Final counts were calculated based on the number of spot-forming cells (SFC) per 10^6^ cells after background subtraction.

Group 2 vaccinated animals (AdgB + AdIE1) were additionally evaluated for cell-mediated response against the IE1 antigen using a previously developed guinea pig IFN-γ ELISPOT assay (48) (**Figure 5A**). An overlapping peptide library for GP123 (IE1) (**Supplementary Table 1**) was used to evaluate splenocytes isolated from vaccinated animals (n = 3). Assays were performed 14 days after the last vaccination. Induction of a cell-mediated response in splenocytes identified three different active peptide pools containing eight to ten 15mer peptides corresponding to GP123. The vaccinated animals demonstrated an immunogenic response to IE1 (**Figure 5A**). The positive GP123 peptide pools are listed in **Supplementary Table 2**. Assays were additionally performed on GPCMV (22122) seropositive and seronegative animals (**Figure 5B**) as positive and negative controls, respectively. Since GPCMV seropositive animals would be exposed to IE1 antigen, there would be an expectation of a positive response to the GP123 peptide antigens in a guinea pig IFN-γ ELISPOT assay, unlike seronegative animals (**Figure 5B**). The cellular response in vaccinated animals was similar to that in GPCMV-infected seropositive control animals. The uninfected seronegative animals did not respond to GP123 peptide stimulation. The results demonstrated that co-vaccination with AdgB + AdIE1 induced an IE1 cell-mediated response in group 2 animals.

### 3.3 Preconception vaccine protection against cCMV

**Figure 6:**
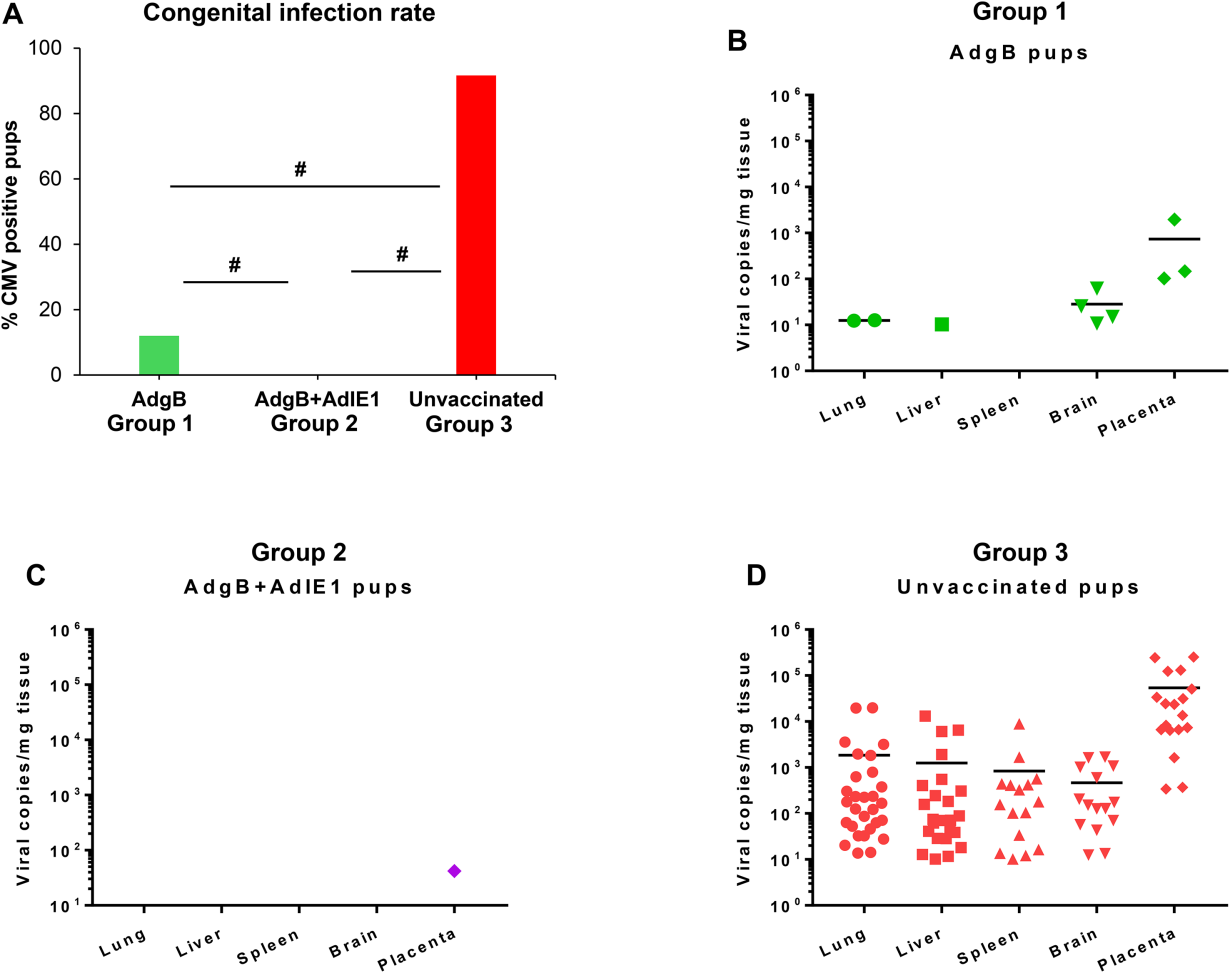
Vaccine protection against dual-strain GPCMV congenital infection. **(A)** Congenital infection rate determined by the percentage of GPCMV-positive pups in AdgB vaccinated (12%, green) or AdgB + AdIE1 vaccinated (0%, purple) compared to unvaccinated (92%, red) pups based on detectable virus in tissues tested by real-time PCR. Statistical analysis was performed using the Student’s t-test # *p <*0.05. Pup viral load in each study group **(B)** AdgB vaccinated (Group 1, green); **(C)** AdgB + AdIE1 vaccinated (Group 2, purple); and **(D)** Unvaccinated group (Group 3, red) with detectable levels of GPCMV found in the lung, liver, spleen, brain, or placental tissues. The total number of detectable samples from each group and the statistical analysis are presented in **Table 2**. The mean values of each test group are represented by a black horizontal line **(B, D)**.

Approximately 4 weeks after the last vaccination, dams were mated with seronegative males. At 30–35 days of pregnancy (late second 2nd trimester), dams were challenged with wild-type GPCMV. At the time of the challenge, the average duration since the last vaccination was approximately 3 months. The challenge virus consisted of two strains of GPCMV (22122 and TAMYC strains), with each strain being separately administered into the opposite flanks of the animal by SQ injection (10^5^ pfu GPCMV per injection). A control group (Group 3) of seronegative unvaccinated dams (n = 10) was mated and similarly challenged with wild-type GPCMV strains during the 2nd trimester. Animals were allowed to proceed to term, and pups from all three groups were evaluated for their viral load. The results are shown in **Tables 1**, **2** and **Figure 6**. A summary table of the congenital CMV study outcomes is shown in **Table 1**, which compares litters from vaccine groups 1 and 2 to those from unvaccinated control group 3 pups. In the control unvaccinated group, 25% (9/36) of animals were stillborn, compared to one stillborn pup in each vaccine group, representing 2% (group 1) and 2.8% (group 2) of the total litters. In the unvaccinated group, 78% of the stillborn pups in the control group were CMV-positive, but none of the stillborn pups in either vaccine group had detectable viruses in their tissue organs. Despite the absence of detectable virus in stillborn pup tissues in the vaccine groups, we assumed that maternal CMV infection was the basis for pup death and not a complication of pregnancy. Additionally, we cannot rule out the possibility of the virus being present in pups below detectable assay levels. Both vaccine groups had approximately similar high percentages of live pups (98% and 97.2% for groups 1 and 2, respectively) compared to 75% live pups in the no-vaccine control group. A comparison of the numbers of CMV-positive live pups was statistically significant between the vaccine groups and no vaccine control (**Table 1**): control vaccine group 3 live pups (96.3% CMV+ pups); AdgB vaccine group 1 (12.2% CMV+ pups); the AdgB + AdIE1 vaccine group 3 (0% CMV+ pups). Furthermore, there was a statistically significant difference in CMV-positive live pups between the two study groups (groups 1 and 2).

**Table 1:** Congenital infection outcomes for live versus stillborn pups.

Study outcome	Group 1: AdgB	Group 2: AdgB + AdIE1	Group 3: Unvaccinated
Pregnant	17/17 (100%)	13/13 (100%)	10/10 (100%)
Litters delivered	17	13	10
Litters with only live pups	16	12	7
Litters with mix (live and dead) pups	1	1	1
Litters with only dead pups	0	0	2
Total pups (live) * Live pups CMV+*	49 (98.0%) *6/49* *^#^ *(12.2%)*	35 (97.2%) 0/35*^#^ *(0.0%)*	27 (75%) *26/27* * *(96.3%)*
Total pups (dead) * Stillbirth pups CMV+*	1 (2.0%) *0/1 (0.0%)*	1 (2.8%) *0/1 (0.0%)*	9 (25%) *7/9 (77.8%)*

*Statistical analysis comparing GPCMV positive pups of live-born in AdgB or AdgB + AdIE1 vaccinated against unvaccinated groups: *p < 0.0001 determined by Fisher exact test.

**^#^** Comparison of GPCMV positive pups of live-born between vaccine groups AdgB vs AdgB + AdIE1groups: #*p <*0.05 determined by Fisher exact test.

**Table 2:** cCMV outcome of pups with detectable virus in organs.

cCMV outcome	Group 1: AdgB*	Group 2: AdgB + AdIE1*	Group 3: Unvaccinated*
CMV+ pups	6/50^#^ (12.0%)	0/36^#^ (0%)	33/36 (91.67%)
Lung	2/50 (4.0%)	0/36 (0.0%)	29/36 (80.56%)
Liver	1/50 (2%)	0/36 (0.0%)	24/36 (66.67%)
Spleen	0/50 (0%)	0/36 (0.0%)	15/36 (41.6%)
Brain	4/50^□^ (8.0%)	0/36^□^ (0.0%)	15/36 (41.6%)
Placenta	3/20 (15%)	1/25 (4.0%)	18/18 (100%)

*Significant difference between each study group (AdgB or AdgB + AdIE1) compared to unvaccinated group in total CMV+ pups and each tissue groups. Statistics determined by Fisher’s exact test *p* < 0.001.

^#^Significant difference in CMV+ pups between AdgB and AdgB + AdIE1 groups determined by Fishers exact test *p <*0.05.

^□^No significant difference in all tissues except in the brain between the two study groups (1 and 2) as determined by z test *p <*0.05.

Overall, both vaccine strategies were highly successful in reducing cCMV transmission rates in pups (**Table 2**). However, the combined gB + IE1 vaccine strategy was the most successful, with 0% detectable virus in all pups, compared to 91.7% of pups positive in the control group 3 (**Table 2**, **Figure 6**). The gB vaccine strategy was also highly effective, with a reduction in cCMV transmission to 12% in pups in group 1 (**Table 2**, **Figure 6**). Both vaccine groups had statistically significant outcomes in terms of the reduction in cCMV transmission. In the no-vaccine control group, three pups showed viral disseminated to all target organs tested (**Table 2**, **Figure 6**), with 41.6% of pups positive for CMV in the brain compared to 8% in the gB vaccine group and 0% in the gB + IE1 vaccine group (**Table 2**). In both vaccine groups, the virus was detected in the placenta at a low level, 1/25 pups (4%) for group 2 and 3/20 (15%) for group 1, but this was a substantial decrease from the level of virus in the placentas of the no-vaccine control group, 18/18 (100%) (**Table 2**, **Figure 6**).

## 4 Discussion

Previous GPCMV vaccine studies have demonstrated the limited ability of gB to provide high-level protection against CMV, especially for cross-strain protection against highly cell-associated clinical GPCMV strain (TAMYC). The inclusion of a T cell antigen can potentially enhance cross-strain protection against cCMV. In HCMV, convalescent patients produce T cell responses against two specific viral proteins (pp65 and IE1) that are significant for vaccine development (45), and GPCMV-infected animals exhibit T cell responses to both IE1 and pp65 homologs (21, 34, 48). Multiple studies with GPCMV pp65 (GP83) or in combination with gB did not enhance cCMV protection against the 22122 strain, as reviewed by Choi and McGregor (12). Indeed, complete protection against cCMV (22122 strain) can be achieved using a live attenuated GPCMV mutant lacking the pp65 antigen (24). Our previous IE1 vaccine study (48) demonstrated the ability of cell-mediated T cell response to provide high level cross-strain protection against cCMV. This study demonstrated the ability of the gB immune response to synergize with the IE1 response in the gB + IE1 vaccine group to further improve cCMV protection, unlike previous gB and pp65 dual-antigen vaccine strategies. Importantly, the gB + IE1 vaccine was cross-strain protective against cCMV, with complete protection. In contrast, the gB vaccine reduced cCMV transmission to 12% compared to 92% in the no vaccine control group (80% reduction). However, the AdgB vaccine lacks cross-strain protection against TAMYC strain challenge in a non-pregnant animal model, with the ability of virus to fully disseminate in vaccinated animals (23). An earlier IE1 vaccine strategy reduced cCMV transmission to 23% demonstrating cross-strain protection (48). A previous Ad vaccine-based GPCMV (22122) study by Inoue et al. (55) with a recombinant AdgB (monomeric) vaccine strategy resulted in a 13% cCMV transmission rate to pups in the gB vaccine group compared to 75% cCMV transmission in a control AdlacZ vaccine group, a reduction in transmission of 62% against GPCMV (22122). This indicates that a trimeric gB used in current study further enhances protection against cCMV compared to monomeric gB. Importantly, in the Inoue study (55), the cCMV transmission rate in the control vaccine group (AdlacZ) was generally similar to that of unvaccinated animals in other 22122 strain cCMV vaccine studies (12). Additionally, Ad-based gB and pp65 vaccine strategies did not protect against TAMYC strain challenge in a non-pregnant model (21, 23). This indicates that the Ad vector platform alone does not contribute to protection against GPCMV but represents a simple and effective strategy for target antigen expression. Indeed, an Ad-based vaccine has demonstrated efficacy against congenital Zika virus in preclinical studies (56) and Ad-based vaccines have been used successfully in the clinic against Ebola virus and SARS-CoV2 (57, 58). In future studies, an oral/intranasal vaccination route is a potentially safer approach (59) and avoids pre-existing immunity to specific Ad virus serotypes as well as the low level risk of blood clots from i/m vaccination (60, 61) and potentially enhances mucosal immunity.

Guinea pig T cell response to IE1 was evaluated using a guinea pig-specific IFNγ ELISPOT assay with 15mer overlapping peptides covering the entire length of GP123 to identify three reactive pools of peptides. Each of the 15mer peptides overlapped on the GP123 protein by 11 amino acids to comprehensively cover potential antigenic peptides, and peptides were used at a concentration of 5 µg/ml, which has been shown to be just as effective as using 9mers at a lower concentration (62). A limitation of the available immunological reagents dedicated to guinea pigs generally prevents the ability to further characterize guinea pig cell-mediated response much beyond IFNγ ELISPOT assays, and additional dedicated guinea pig T cell assays need to be developed. Studies in transplant patients suggest the importance of CD8^+^ T cell response against IE1 for protection from CMV (63), and our assumption is that the AdIE1 vaccine elicits a CD8^+^ T cell response. HCMV (Towne) vaccination of seronegative patients resulted in IE1 CD8^+^ T cell response but not pp65 (64). A HCMV Toledo/Towne vaccine similarly induced an IE-focused response (65). Additionally, CD8^+^ T cell IE1 response, but not pp65 response, is associated with protection against HCMV in solid organ transplant patients (63). HCMV studies in human placental decidual tissue and resident T cells suggest the importance of HCMV specific resident CD8^+^ T cells for protection against HCMV (66). Evaluation of resident guinea pig placental T cells would likely be informative, especially if vaccine responses were contrasted with natural convalescent immunity. Future GPCMV research should ideally better define CD4^+^ and CD8^+^ T cell responses to GPCMV IE1 and other viral antigens. A transcriptomics approach applied to HCMV clinical studies that enables the characterization of signature T cell responses to an HCMV vaccine could be applied to GPCMV vaccines (67). Recent in-depth sequencing of the Hartley strain guinea pig genome has enabled an RNA-seq transcriptomics approach in guinea pigs (68). This strategy has recently been successfully applied to guinea pig CD4^+^ and CD8^+^ T cell studies in HSV vaccine research (69).

A limitation of previous gB vaccine studies in the guinea pig model is that the majority of gB antigens studied lacked the ability to form a trimeric gB complex, which enhances the level of neutralizing antibody response against GPCMV (20). Although a trimeric gB vaccine was highly effective against the 22122 strain in a horizontal (SQ) challenge model, it lacked cross-strain protection against GPCMV (TAMYC) dissemination (SQ) despite 99% similarity in the gB amino acid sequence between strains (20, 21). This demonstrates the limitation of gB cross-strain protection despite success against cCMV with approximately 50% protection against cCMV (22122) in subunit-and vector-based gB vaccine strategies, as reviewed by Choi and McGregor (12). The immunological basis for the limitations of prior GPCMV gB vaccine strategies in the guinea pig model is poorly defined. In this report, additional aspects of the gB immune response were characterized between vaccine groups and in comparison to natural convalescent GPCMV immunity (22122 strain) using novel gB-specific ELISAs, which included prefusion gB and gB(AD1, as well as established gB and anti-GPCMV ELISAs (20). In HCMV, the gB AD1 domain is necessary for gB oligomerization (53), and this is presumably the case for GPCMV. HCMV gB AD1 domain antibody response is common in all convalescent patients, and AD1 is also a major target for neutralizing antibodies (70–72). It is interesting to note that in HCMV clinical trials for both gB/MF59 and Moderna mRNA-1647 vaccines, gB AD1 binding antibodies were not detected (73, 74). In the current GPCMV vaccine study, all vaccinated animals had a specific response to gB(AD1) antigen, as did convalescent sera (22122 strain) (**Figure 1**). Both vaccine groups induced a higher titer anti-gB response than natural immunity, with the gB vaccine group inducing approximately 2-fold higher titer response than the gB + IE1 vaccine group; however, individual responses were more widespread (**Figure 3**). In contrast, the AD1 immune response in both vaccine groups was more tightly clustered, with both groups having statistically higher titers than natural convalescent immunity. Animals received identical dosages (titers) of Ad vectors encoding either IE1 or gB. Previous studies with a 2-fold increase in the Ad vector did not result in modified outcomes for the immune response. Indeed, multiple vaccinations are required to induce the required response (i.e., repeated exposure to antigens over time, and a single high dose is insufficient). Therefore, it seems unlikely that this is a factor since the outcomes are so contrasting.

HCMV gB is a fusogenic protein that has the potential to exist in a pre-fusion confirmation on viral particles, which might be more effective as a vaccine target, and a locked version of HCMV pre-fusion gB was recently described (52, 75). Based on the alignment of the HCMV and GPCMV gB amino acid sequences, similar modifications were made to the GPCMV gB ORF to generate a synthetic gene encoding a locked prefusion gB. Both vaccine groups exhibited a response to the GPCMV prefusion gB antigen, with antibody titers statistically higher than natural immunity; however, the gB + IE1 vaccine group had a higher response than the gB-only vaccine group. The results suggest that the presence of IE1 antigen did not affect the gB immune response to the AD1 domain despite lower anti-gB titers, but that inclusion of IE1 antigen resulted in higher levels of anti-prefusion gB response. This may have contributed to the enhanced protection against cCMV in the gB + IE1 vaccine group compared to the gB group. In the case of one litter (dam#3) in the gB vaccine group, the differential antibody immune response might have contributed to placental and pup infection. In this litter (dam#3), 2/3 pups and 2/3 placenta were infected with the virus, the dam had a higher anti-gB titer (20,480), and the highest gB(AD1) titer (5,120) but a lower prefusion gB titer (1,280) than the mean value (**Figure 3**). This suggests that neither a high AD1 nor a high gB antibody titer is a predictor of a positive outcome against viral challenge. In contrast, a low response to the pre-fusion gB antigen might indicate a greater risk of cCMV. However, this aspect likely requires further evaluation of both AD1 and prefusion gB immune responses, which are beyond the scope of this initial research. Several antigenic domains exist for HCMV gB (76), and this is likely the case for GPCMV as well. Future studies should also characterize potential additional homolog antigenic domains, especially a homolog N-terminal AD2 domain, where only 50% of HCMV convalescent patients have an antibody response to this neutralizing antibody domain (76, 77).

In HCMV, antibodies to gB as well as non-neutralizing antibodies to other viral proteins can provide protection by ADCC and ADCP pathways (73, 78, 79); however, the evaluation of this aspect of the immune response is missing in guinea pig studies. Most certainly, GPCMV monomeric gB, in comparison to trimeric gB, produces a high level of non-neutralizing antibodies (20), and control of infection by ADCC/ADCP pathways is perhaps a realistic possibility. However, in animals, a gB vaccine lacks cross-strain protection against a highly cell-associated clinical TAMYC strain, suggesting a limitation of this antibody cell-associated immune response in guinea pigs (23). This is also suggested by the fact that natural GPCMV (22122) convalescent immunity with both neutralizing and non-neutralizing antibodies to various viral antigens fails to prevent infection by the heterologous TAMYC strain challenge (**Figure 1**). Although antibody-based ADCC effects may enable the targeting of infected cells, HCMV has the capacity to evade ADCC induction (78–80). Consequently, the importance of ADCC/ADCP pathways in contributing to protection against GPCMV is ambiguous, especially if the virus has the capacity to evade these pathways, as suggested by the ability of clinical strain TAMYC to evade both gB and GPCMV DISC vaccines, which are highly effective against 22122 strain (23, 24). Furthermore, temporary complement depletion in an AdgB non-pregnant animal model vaccine study demonstrated that protection against GPCMV (22122 strain) was unaffected by complement depletion in vaccinated guinea pigs (20).

An additional important aspect of the antibody response that remains to be evaluated is fetal protection by transplacental transfer of protective maternal antibodies to the fetus *in utero* (81). In guinea pigs, both human and guinea pig IgG can be transferred across the placenta (82). Previously, anti-gB passive antibody therapy in guinea pigs demonstrated partial placental and fetal protection against GPCMV, suggesting that this is a potential protective strategy; however, transplacental transfer of gB antibodies to the fetus remains to be more fully investigated (83). Future evaluation of this model of transplacental transfer of antibodies is merited by recent research, which indicates that HCMV antibodies in humans can activate fetal NK cells via Fc receptors, enabling the cytotoxic targeting of virus-infected cells (84). Consequently, it is important to determine in future research whether this mechanism exists in the guinea pig model to improve the translational impact of GPCMV vaccine studies. Potentially, GPCMV gB protein is a T cell target antigen based on studies with HCMV gB in convalescent patients (85), and the possible contribution of cell-mediated T cell response to vaccine protection should be evaluated in future GPCMV gB research. However, as previously demonstrated, the lack of cross-strain protection of an AdgB vaccine against the highly cell-associated TAMYC virus with 99% amino acid gB identity between strains suggests that a T cell response to gB has minimal protective impact compared to an antibody response (23).

Similar to HCMV, GPCMV has two pathways of cell entry, direct and endocytic, both of which require gB. However, additional gH/gL-based glycoprotein complexes and specific cell receptors are necessary for each entry pathway (86). Similar to HCMV, GPCMV direct cell entry requires the cellular receptor PDGFRA and interaction with triplex gH/gL/gO (36). The cell types that are mainly positive for PDGFRA are fibroblasts, but placental trophoblast cells can be positive or negative for PDGFRA (87). Endothelial and epithelial cells tend to be negative for PDGFRA and endocytic infection requires the PC in both HCMV and GPCMV (17, 22, 86). In HCMV, PC interacts with various cell receptors, but NRP2 is the most common cell receptor in epithelial and endothelial cells (88). Most recently, GPCMV PC was demonstrated to interact with guinea pig NRP2 for endocytic cell entry, and CRISPR-based knockout of this receptor blocked endocytic cell entry (89). PC is important for GPCMV pathogenicity and cCMV; however, low-level fetal transmission can occur in the absence of PC (17, 18, 35). Importantly, PC has gained significant attention as a candidate HCMV antigen against cCMV (90) with enhanced virus neutralization (91). Recent GPCMV studies have demonstrated vaccine success against cCMV (22122 strain) by including PC as a viral antigen (19, 92); however, cross-strain vaccine protection is lacking against the clinical TAMYC strain (24). This report suggests that cross-strain cCMV vaccine protection can be achieved without the inclusion of PC antigens. A limitation of the current study is that virus challenge was by SQ route and did not evaluate horizontal animal-to-animal transmission. The additional inclusion of PC antigen in vaccine design would potentially increase mucosal protection against oral/nasal routes of virus infection, which would be an important aspect of future CMV vaccine research in this model.

## 5 Limitations of study

This preclinical animal model study demonstrates the feasibility of a cross-strain protective vaccine against cCMV, which has been an elusive goal despite over 50 years of CMV vaccine research. Importantly, cCMV protection in guinea pigs can be achieved by a vaccine targeting key functional homolog viral proteins: (1) gB, essential for virus entry into all cell types, and (2) IE1, essential for lytic virus replication. However, this study has several limitations that require future research to provide further information on the basis for vaccine protection against cCMV. Although this report provides novel insights into the development or use of antibody ELISAs for specific aspects of the GPCMV gB immune response (trimeric gB, AD1, and pre-fusion gB), there is no evaluation of the immune response to other potential gB subdomains apart from the AD1 oligomerizing homolog domain. Currently, it is unknown whether GPCMV gB encodes additional HCMV homolog immunogenic subdomains and whether responses to these domains vary between viral strains or gB vaccines. However, the demonstration of a response to pre-fusion gB is potentially a key insight into vaccine efficacy. Although neutralizing antibody assays are performed on fibroblasts and epithelial cells for both GPCMV strains, there has been no evaluation of ADCC or ADCP gB antibody effects, which have been demonstrated to be important in controlling HCMV infection. Currently, these assays have not been developed for guinea pigs, which prevents evaluation at present time; however, in a previous study, temporary depletion of complement had a limited impact on gB vaccine efficacy (20). Additionally, there was no evaluation of maternal–fetal antibody transfer, which might provide vaccine efficacy to the fetus in the *in utero* state. However, evaluation of the placenta suggested that the vaccine strategy was effective in preventing GPCMV placental infection, which would have to occur prior to viral infection of the fetus. At present, it is unknown whether GPCMV gB evokes a T cell response and whether this contributes to vaccine efficacy in addition to the demonstrated protective vaccine antibody response. Furthermore, GPCMV IE1 cell-mediated immune response studies are restricted to an IFN-gamma ELISPOT assay. Consequently, there is a lack of a comprehensive evaluation of the T cell response to IE1 in guinea pigs and whether the vaccine response differs from that of natural convalescent GPCMV infection immunity. Overall, the limitations described are mainly due to the limited availability of guinea pig reagents/assays, which severely reduces the scope of immunological studies that can be performed with this model. However, as noted in the discussion section, approaches and assays are being developed to improve the immunological insight of this model.

## 6 Conclusion summary

This report demonstrates that a combined Ad-based CMV vaccine strategy of gB and IE1 provides complete cCMV protection against both the prototype 22122 strain and a novel clinical strain of GPCMV (TAMYC). There is a risk of cCMV from both primary and non-primary infections with a new CMV strain. Consequently, a vaccine strategy needs to provide high efficacy and cross-strain protection. Complete cross-strain protection against cCMV is an important milestone in this model. The results suggest that a combined approach of CMV antibody (gB) and T cell (IE1) antigen vaccine candidates is an important foundational strategy and would be highly protective against HCMV. However, an HCMV vaccine would require the modification of IE1 to attenuate functional activity and improve vaccine safety. These results suggest that an antibody response against viral PC is not absolutely required for protection against cCMV. However, a limitation of the current study is that it did not evaluate horizontal animal-to-animal transmission, where the inclusion of PC antigen in vaccine design could potentially increase mucosal protection against oral/nasal routes of GPCMV infection in future studies.

## Data Availability

The original contributions presented in the study are included in the article/**Supplementary Material**. Further inquiries can be directed to the corresponding author.
