# Supplementary material for: Complete cross strain protection against congenital cytomegalovirus infection requires a vaccine encoding key antibody (gB) and T-cell (immediate early 1 protein) viral antigens
Source: Front Immunol. 2025 Oct 22;16:1649656. doi: 10.3389/fimmu.2025.1649656 (PMC12586184; doi:10.3389/fimmu.2025.1649656)
Supplement: Supplementary file 1 [file DataSheet1.pdf]

Supplementary Material

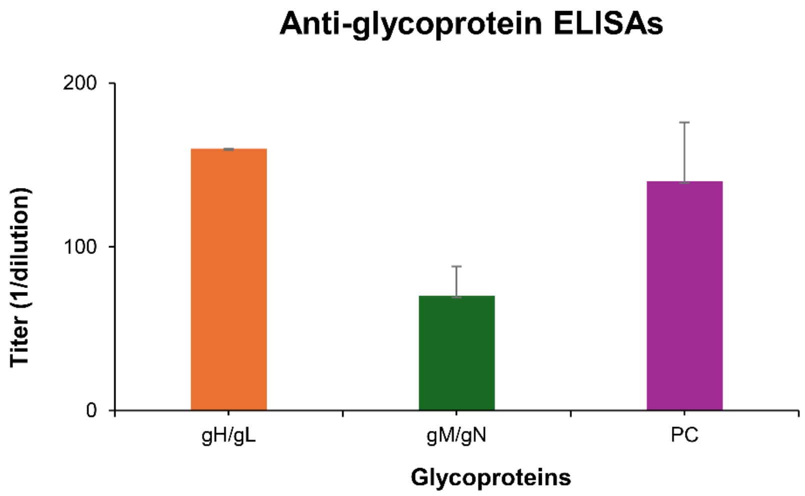

Supplementary Figure 1. Immune response to single shot of 22122 strain GPCMV. Specific viral glycoprotein complex ELISA assays were conducted on pooled sera from seropositive GPCMV(22122) infected animals and used to determine to determine ELISA titers for: anti-gHgL (orange); anti-gMgN (green); or anti-PC (purple) immune titers. Anti-GPCMV and anti-gB results are shown in Figure 3.

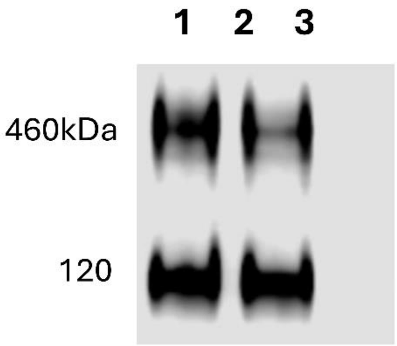

Supplementary Figure 2. Western blot analysis of GPCMV prefusion gB expression. Prefusion gB transfected GPL cell lysate detected with anti-FLAG antibody (1/1000). Lane 1 & 2 prefgB plasmid GPL transfected cell monolayer total cell lysate at 24 hr post transfection; Lane 3 mock infected GPL cell lysate. SDS-PAGE carried out under non-denaturing conditions as described in materials and methods. Monomer prefgB (120 kDa) and triplex gB (460 kDa) indicated.

A

```
GPCMV  489  CLDQKRTAEVLHELSKINPSNILSAIFGVPVAARVVGDVISLAKCIEVNQSTVLIKGDMR  548
            C+DQ+RT EV  ELSKINPS ILSAI+   P+AAR +GDV+ LA C+ +NQ++V +   DM
HCMV   504  CVDQRRTLEVFKELSKINPSAILSAIYNKPIAARFMGDVLGLASCVTINQTSVKVLRDMN  563

GPCMV  549  KFSDDGKLEGCYSRPVVWFSMKNSTEVRLGQLGEDNEILLGTHRMETCQTQDYRIFVAGD  608
                 G+    CYSRPVV F+  NS+ V+ GQLGEDNEILLG HR E CQ     +IF+AG+
HCMV   564  VKESPGR---CYSRPVVIFNFVNSSYVQYGQLGEDNEILLGNHRTEECQFPSLKIFIAGN  620

GPCMV  609  IGYEFQQYVFTKKINLSEIDIIDTMIALKTEPLENIDFKVLELYSRDELAQANVFDLESI  668
              YE+   Y+F + I+LS I   +D+MIAL   +PLEN DF+VLELYS+ EL   +NVFDLE I
HCMV   621  SAYEYVDYLFKRMIDLSSISTVDSMIALDIDPLENTDFRVLELYSQKELRSSNVFDLEEI  680
```

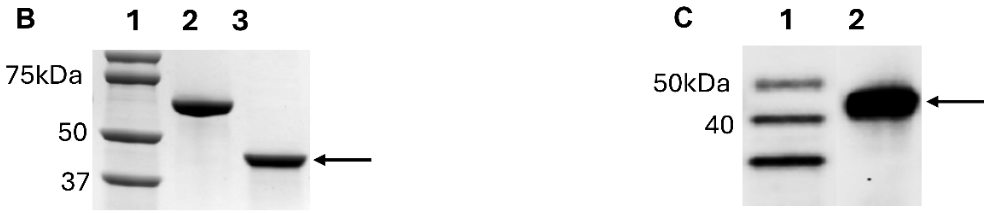

Supplementary Figure 3. GPCMV gB(AD1) analysis. (A) Protein BLAST alignment of HCMV gB AD1 compared to GPCMV gB AD1 region. Commercial HCMV (Towne) GSTgB(AD1) protein (Sigma), region 549-650 highlighted in blue. Aligned HCMV/GPCMV gB AD1 region as described by Britt et al., 2005 [53]. Underlined sequence GPCMV gB AD1 extended region used for generation of GPCMV GST-gB(AD1) fusion ORF with similar flanking sequence to commercial (Sigma) HCMV gB(AD1) fusion GST protein . (B) SDS PAGE gel of purified recombinant GPCMV GST-gB(AD1) protein (Genscript). Lane 1 marker; lane 2 BSA protein control; 3 GST-gB(AD1) protein resolved by Coomassie blue staining. (C) Western blot of recombinant GPCMV GST-gB(AD1) protein detected with anti-GST antibody. Lane 1 kDa size markers; lane 2 GST-gBAD1 protein.

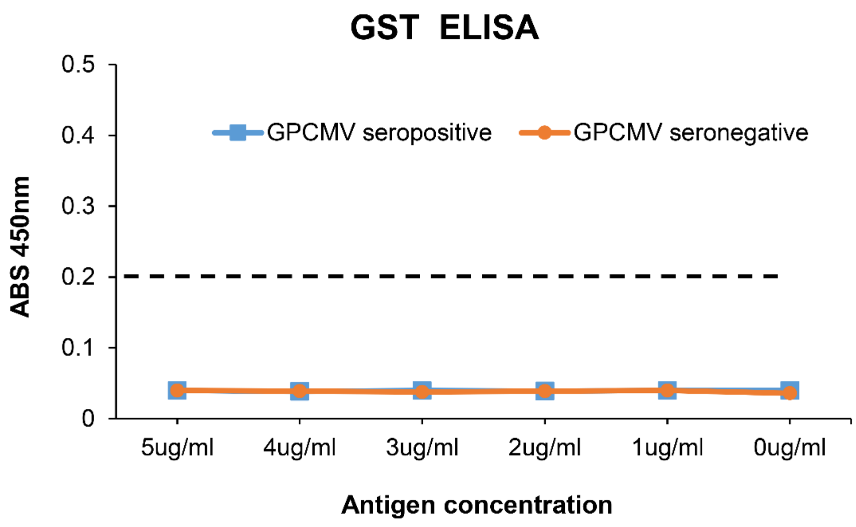

Supplementary Figure 4. Antibody immune response to control GST antigen for GPCMV seropositive and seronegative sera. Varying concentration of control carrier recombinant GST protein was used as coating antigen (0ug/ml to 5ug/ml) to test the reactivity of GPCMV seropositive and seronegative sera to GST. Assay negative cutoff for standard GPCMV ELISA assay, ABS 450nm ≤ 0.2, black dash line.

Supplementary Table 1.

List of individual GP123 peptides (15mers) used to generate peptide pools for the IFNγ-ELISPOT assay.

RALLLVMKRSIQELQ	SHSDHFDTIILKALK	GRRDYYFCDVFSRLR	NGVRPSSAFLQCLPS
LVMKRSIQELQDSLR	HFDTIILKALKSNLD	YYFCDVFSRLRTDIA	PSSAFLQCLPSYLRN
RSIQELQDSLRQHRE	IILKALKSNLDAIRN	DVFSRLRTDIAESVT	FLQCLPSYLRNKYAA
ELQDSLRQHRELAWE	ALKSNLDAIRNDLVI	RLRTDIAESVTSSFD	LPSYLRNKYAALLPP
SLRQHRELAWEECKR	NLDAIRNDLVIETRE	DIAESVTSSFDAMDR	LRNKYAALLPPLNTP
HRELAWEECKRCIDN	IRNDLVIETREMVLQ	SVTSSFDAMDRDFNS	YAALLPPLNTPITGP
AWEECKRCIDNVWVR	LVIETREMVLQRWLH	SFDAMDRDFNSKLFM	LPPLNTPITGPMSES
CKRCIDNVWVRLQRH	TREMVLQRWLHYTSF	MDRDFNSKLFMAITR	NTPITGPMSESEGAS
IDNVWVRLQRHERVT	VLQRWLHYTSFCDVP	FNSKLFMAITRHTQY	TGPMSESEGASGRLR
WVRLQRHERVTDSIF	WLHYTSFCDVPLHNH	LFMAITRHTQYLDAV	SESEGASGRLRPRPR
QRHERVTDSIFNANK	TSFCDVPLHNHNLRT	ITRHTQYLDAVNAFI	GASGRLRPRPRKRKA
RVTDSIFNANKQACS	DVPLHNHNLRTAACF	TQYLDAVNAFIIEEV	RLRPRPRKRKADGTM
SIFNANKQACSMVQG	HNHNLRTAACFINNL	DAVNAFIIEEVLRQF	RPRKRKADGTMLTRA
ANKQACSMVQGMKRM	LRTAACFINNLKSGT	AFIIEEVLRQFTWGQ	RKADGTMLTRAKVAE
ACSMVQGMKRMLKNY	ACFINNLKSGTWFSD	EEVLRQFTWGQTDPS	GTMLTRAKVAELAGE
VQGMKRMLKNYEAMT	NNLKSGTWFSDAQVK	RQFTWGQTDPSLIVQ	TRAKVAELAGEPSTS
KRMLKNYEAMTPYAV	SGTWFSDAQVKTVLT	WGQTDPSLIVQCLLP	VAELAGEPSTSNMAD
KNYEAMTPYAVVQSA	FSDAQVKTVLTLIEL	DPSLIVQCLLPVIEK	AGEPSTSNMADSGDE
AMTPYAVVQSALRDI	QVKTVLTLIELLVDD	IVQCLLPVIEKALSD	STSNMADSGDETQLD
YAVVQSALRDIASHS	VLTLIELLVDDVGRR	LLPVIEKALSDLNGV	MADSGDETQLDEDIY
QSALRDIASHSDHFD	IELLVDDVGRRDYYF	IEKALSDLNGVRPSS	GDETQLDEDIYTTE
RDIASHSDHFDTIIL	VDDVGRRDYYFCDVF	LSDLNGVRPSSAFLQ	

Supplementary Table 2.

Peptide sequences of reactive pools used in guinea pig IFNγ-ELISPOT assay against GP123.

Pool IV	Pool VI	Pool XVI
ELQDSLRQHRELAWE	HRELAWEECKRCIDN	SFDAMDRDFNSKLFM
ANKQACSMVQGMKRM	VQGMKRMLKNYEAMT	MDRDFNSKLFMAITR
HFDTIILKALKSNLD	ALKSNLDAIRNDLVI	FNSKLFMAITRHTQY
DVPLHNHNLRTAACF	LRTAACFINNLKSGT	LFMAITRHTQYLDAV
VDDVGRRDYYFCDVF	YYFCDVFSRLRTDIA	ITRHTQYLDAVNAFI
LFMAITRHTQYLDAV	TQYLDAVNAFIIEEV	TQYLDAVNAFIIEEV
LLPVIEKALSDLNGV	LSDLNGVRPSSAFLQ	DAVNAFIIEEVLRQF
NTPITGPMSESEGAS	SESEGASGRLRPRPR	AFIIEEVLRQFTWGQ
AGEPSTSNMADSGDE	MADSGDETQLDEDIY	EEVLRQFTWGQTDPS
		RQFTWGQTDPSLIVQ
